# Long-term outcomes of definitive chemoradiotherapy with docetaxel, cisplatin, and 5-fluorouracil for postoperative locoregional recurrent esophageal cancer: a prospective phase II study

**DOI:** 10.3389/fonc.2026.1868181

**Published:** 2026-07-09

**Authors:** Keiichi Jingu, Rei Umezawa, Takaya Yamamoto, Noriyoshi Takahashi, Yu Suzuki, Keita Kishida, So Omata, Hinako Harada, Masanobu Takahashi, Takashi Kamei, Haruo Matsushita

**Affiliations:** 1 Department of Radiation Oncology, Tohoku University Graduate School of Medicine, Sendai, Japan; 2 Department of Clinical Oncology, Tohoku University Graduate School of Medicine, Sendai, Japan; 3 Department of Surgery, Tohoku University Graduate School of Medicine, Sendai, Japan

**Keywords:** chemoradiotherapy, DCF-RT, phase II study, postoperative recurrent esophageal cancer, radiotherapy

## Abstract

**Background:**

Postoperative locoregional recurrence of esophageal cancer remains a major clinical challenge, with no established standard salvage treatment. Although definitive chemoradiotherapy (CRT) is widely used, outcomes with conventional platinum plus 5-fluorouracil (FP) regimens remain unsatisfactory. This phase II study aimed to evaluate the efficacy and safety of CRT combined with docetaxel, cisplatin, and 5-fluorouracil (DCF-RT).

**Methods:**

In this single-arm, prospective phase II trial, 29 patients with postoperative locoregional recurrent esophageal cancer without distant metastasis were enrolled between 2015 and 2023. All patients received radiotherapy (60 Gy in 30 fractions) with concurrent DCF chemotherapy. The primary endpoint was progression-free survival (PFS), compared with a historical control of 12 months. Secondary endpoints included overall survival (OS) and treatment-related toxicities.

**Results:**

With a median observation period for survivors of 77.5 months, the median PFS was 48.0 months, substantially exceeding the predefined threshold. The 3-year and 5-year PFS rates were 55.2% and 49.7%, respectively. The 3-year and 5-year OS rates were 75.2% and 58.8%, respectively. Treatment compliance was high, with 89.7% of patients completing the planned regimen without dose reduction. Grade 4 hematologic toxicities occurred in 3 patients, and one patient developed grade 3 heart failure. No grade ≥2 late adverse events were observed.

**Conclusions:**

DCF-based chemoradiotherapy demonstrated durable disease control and a favorable safety profile in patients with postoperative locoregional recurrent esophageal cancer. Given the substantial improvement in long-term survival compared with historical outcomes, this regimen may represent a promising curative-intent salvage strategy. Further validation in randomized controlled trials is warranted.

## 1 Introduction

Neoadjuvant chemotherapy and chemoradiotherapy followed by extended radical esophagectomy with two-field or three-field (neck, mediastinum, and abdomen) lymph node dissection have improved the prognosis of patients with esophageal cancer. However, there is recurrence in 30-50% of patients who have undergone an operation and there is locoregional recurrence in 40-55% of patients with postoperative recurrence (1–4). The efficacy and safety of chemoradiotherapy for postoperative locoregional recurrent esophageal cancer have been reported (5–10), mainly from Japanese or Chinese institutions including our institution, and the survival outcome has been improved. In 2012, we reported the results of a prospective study of radiotherapy (RT) and concurrent chemotherapy [nedaplatin and 5-fluorouracil (5-FU)] for postoperative recurrent esophageal cancer with a median observation period of 72 months (11). Definitive chemoradiotherapy (CRT) represents the only potentially curative option for these patients; however, outcomes with conventional platinum plus 5-FU regimens remain suboptimal, with median progression-free survival of approximately 12 months, which is not sufficient. Recently, Docetaxel (DOC)-containing regimens have demonstrated enhanced radiosensitizing effects and improved outcomes in primary esophageal cancer. We therefore conducted a prospective phase II trial to evaluate whether RT with DOC, cisplatin (CDDP) and 5-FU could improve outcomes in the salvage setting.

## 2 Materials and methods

### 2.1 Trial design and patient selection

This open-label, single-arm, phase 2 study was performed between 2015 and 2023 in our hospital according to the following protocol.

Patient selection criteria included 1) 20 to 75 years of age, 2) Eastern Cooperative Oncology Group (ECOG) performance status of 0 to 2, 3) no other active cancer for at least 3 years, 4) no severe cardiac, liver, kidney, mental or pulmonary disease, 5) creatinine clearance ≥ 60 ml/min, 6) adequate bone marrow function, including leukocyte count ≥ 3000/μl, hemoglobin ≥ 10 g/dl and platelet count ≥ 100, 000/μl, 7) locoregional recurrence (including supraclavicular lymph node metastasis) without distant metastasis after no residual tumor (R0) resection by radical esophagectomy with two or three-field lymph node dissection, and 8) no previous therapy for recurrence.

Recurrence was diagnosed comprehensively by upper gastrointestinal endoscopy, ultrasonography, computed tomography (CT) and positron emission tomography using ^18^F-fluorodeoxyglucose (FDG-PET), physical findings and/or cytology.

Exclusion criteria were 1) history of RT around the recurrent lesions, 2) recurrence after endoscopic mucosal resection, 3) second primary cancer in the residual cervical esophagus, 4) presence of severe diabetes or collagen disease, 5) presence of acute inflammatory disease and 6) implantation of a pacemaker or defibrillator.

### 2.2 Treatment

#### 2.2.1 Radiotherapy

All patients were treated with involved-field radiotherapy (IFRT) using three-dimensional conformal radiotherapy (3D-CRT). The target volume was localized for RT in all patients by CT planning. The daily fractional dose of RT was 2.0 Gy, administered 5 days a week, and the total dose was 60.0 Gy. Clinical target volume (CTV) was the addition of a 0.5-1.0-cm margin to the gross target volume (GTV), but areas anatomically unlikely to have tumor invasion were removed. Planning target volume (PTV) was the addition of 0.5-1.0 cm in the axial plane and 1.0-2.0 cm in the cranio-caudal directions to CTV. The dose was prescribed to or near the center of PTV. The normal tissue dose constraints were as follows: mean dose of whole heart <40 Gy, V5 of bilateral lungs <50%, V20 of bilateral lungs <20%, Dmax of spinal cord <48 Gy and Dmax of gastric tube ≤60 Gy. RT was discontinued immediately if grade ≥4 non-hematological toxicity other than radiation-induced esophagitis occurred. When grade ≥4 radiation-induced esophagitis occurred, RT was suspended until it had recovered to grade 2 or lower. If grade ≥4 hematological toxicities occurred, RT was suspended until they were recovered to grade 2 or lower.

#### 2.2.2 Chemotherapy

**Figure 1:**
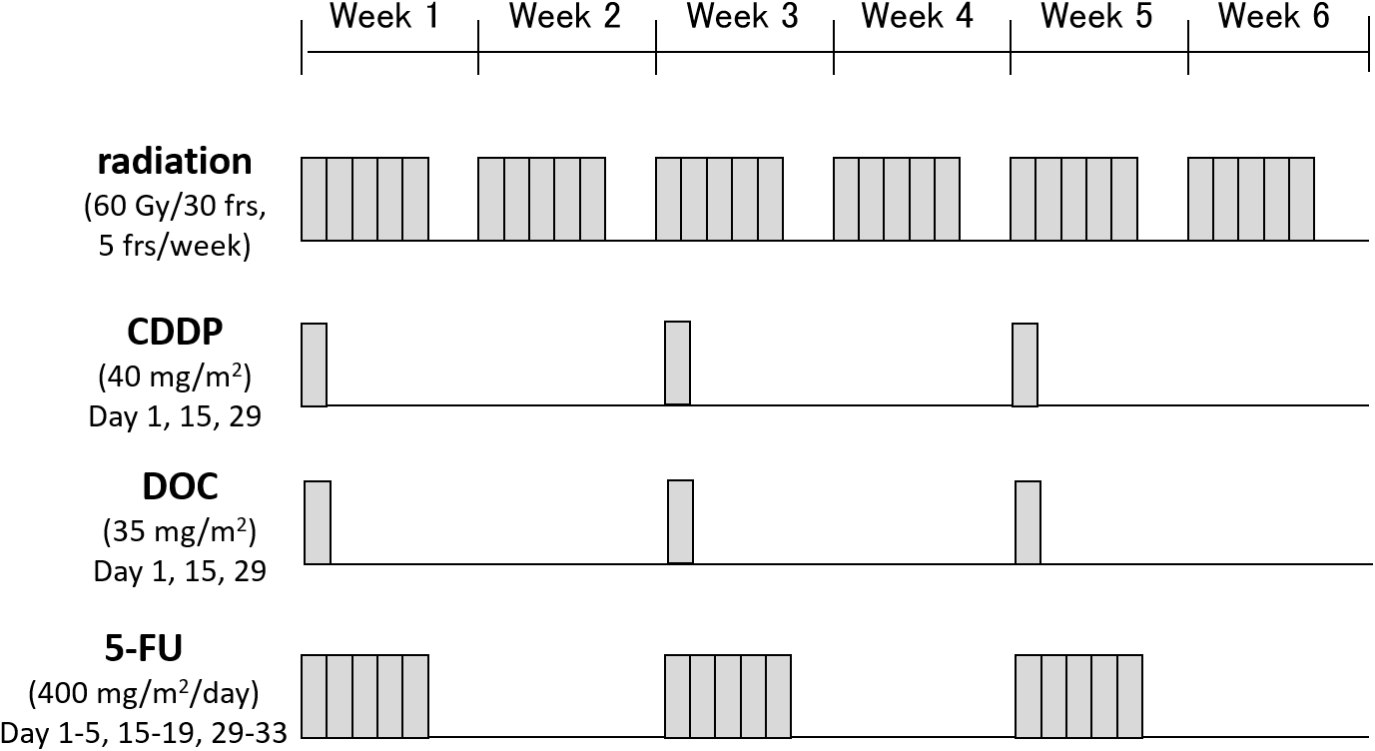
Schedule of the protocol of chemoradiotherapy. Radiotherapy was delivered at 2 Gy per fraction, 5 days per week, for 6 consecutive weeks to a total dose of 60 Gy. Cisplatin (40 mg/m²) and docetaxel (35 mg/m²) were administered on days 1, 15, and 29. Continuous infusion of 5-fluorouracil (400 mg/m²/day) was administered on days 1–5, 15–19, and 29–33.

Each cycle of chemotherapy consisted of 60-minute infusion of DOC at 35 mg/m^2^, 120-minute infusion of CDDP at 40 mg/m^2^ and a 5-day period of 5-FU at 400 mg/m^2^/day (Level 0). This cycle of chemotherapy was repeated every 2 weeks for a total RT dose of 60 Gy (**Figure 1**). Administration of all chemotherapy agents was discontinued until adverse events improved as follows: leukocyte count ≥2, 000/mm^3^; platelet count ≥75, 000/mm^3^; total bilirubin <2.5 mg/dL; serum creatinine ≤1.5 mg/dL; grade 2 or lower nausea, anorexia, diarrhea; and grade 1 or lower pneumonitis.

The doses were reduced to the dose of Level 1 in the subsequent course after at least one of the following toxicities was observed: leukocyte count <2000/mm^3^, platelet count <50000/mm^3^, total bilirubin >3.0 mg/dl, and aspartate aminotransferase or alanine aminotransferase >200 IU/l. If serum creatinine level was between 1.0 and 1.5 mg/dL on the day of initiation or the day before initiation of chemotherapy or serum creatinine was ≥2.0 mg/dL at least once, only the dose of CDDP was reduced to the dose of Level 1. If there were both of the above conditions, CDDP was not administered and only DOC and 5-FU were administered at the doses of Level 1. Even if the dose at Level 1 was used in the second cycle, the third cycle was not performed if any of the above adverse events occurred (Level 2).

Level 0: DOC 35 mg/m^2^, CDDP 40mg/m^2^ and 5-FU 400 mg/m^2^/day

Level 1: DOC 30 mg/m^2^, CDDP 30mg/m^2^ and 5-FU 300 mg/m^2^/day

Level 2: none

The chemotherapy regimen was terminated when patients refused to continue or recovery from adverse events delayed the initiation of the second or third cycle by >2 weeks from the planned schedule.

Completion of the regimen in this study was defined as completion of 3 cycles of DOC + CDDP + 5-FU for a total RT dose of 60 Gy (**Figure 1**).

Any additional therapy after the above chemoradiotherapy was not permitted until re-relapse was confirmed.

### 2.3 Study endpoint

The primary endpoint of the present study was progression-free survival period, and the secondary endpoints were overall survival rate and adverse events. Survival estimate was calculated from the start of radiotherapy to the first identification of disease progression or death. Only progression disease (PD) according to Response Evaluation Criteria In Solid Tumors (RECIST) was defined as failure of the present regimen (re-relapse).

### 2.4 Sample size

In this phase 2 study, a superiority trial was designed to detect 12.0 months or more prolongation in the progression-free survival period, favoring DOC+CDDP+5-FU +RT to historical results of CDDP + 5-FU +RT. The historical median PFS of 12.0 months used for sample size calculation was derived from our previous prospective phase II study of nedaplatin plus 5-fluorouracil with radiotherapy and from published contemporary studies of platinum/5-fluorouracil-based chemoradiotherapy for postoperative locoregional recurrent esophageal cancer available at the time of study design. This would equate to an improvement in progression-free survival period from 12.0 months with CDDP + 5-FU +RT to 24.0 months with DOC+CDDP+5-FU +RT, which was deemed by the study team at the time of study design as a clinically meaningful improvement. The required sample size was determined using the method for single-arm survival studies using transformations of the Kaplan–Meier estimator (12). Assuming a historical median progression-free survival of 12 months, an expected median progression-free survival of 24 months, a one-sided α of 0.1, and 80% power, 25 patients were required.

### 2.5 Follow-up

Follow-up evaluations were performed every 3–6 months for the first 2 years and every 12 months thereafter by endoscopy and CT.

### 2.6 Adverse events

Adverse events were graded according to the Common Terminology Criteria for Adverse Events (CTCAE) v4.0. Acute adverse events were assessed up to 90 days after RT, and late adverse events were assessed afterward.

### 2.7 Statistics

Survival estimates were calculated using the Kaplan-Meier method from the first date of radiotherapy. For exploratory prognostic factor analyses, survival differences were evaluated using the log-rank test. A p value of less than 0.05 was considered significant. All analyses were performed using SPSS 26.0.

### 2.8 Ethics

All procedures were performed in accordance with the principles of the 1964 Declaration of Helsinki and its later amendments or comparable ethical standards. The present study protocol was reviewed and approved by our institutional review board, and informed consent was obtained from each patient before conducting the treatment. The trial was registered with University Hospital Medical Information Network (UMIN) Clinical Trials Registry (UMIN000019269) and Japan Registry of Clinical Trials (jRCTs021200009).

## 3 Results

### 3.1 Patient and tumor characteristics

**Figure 2:**
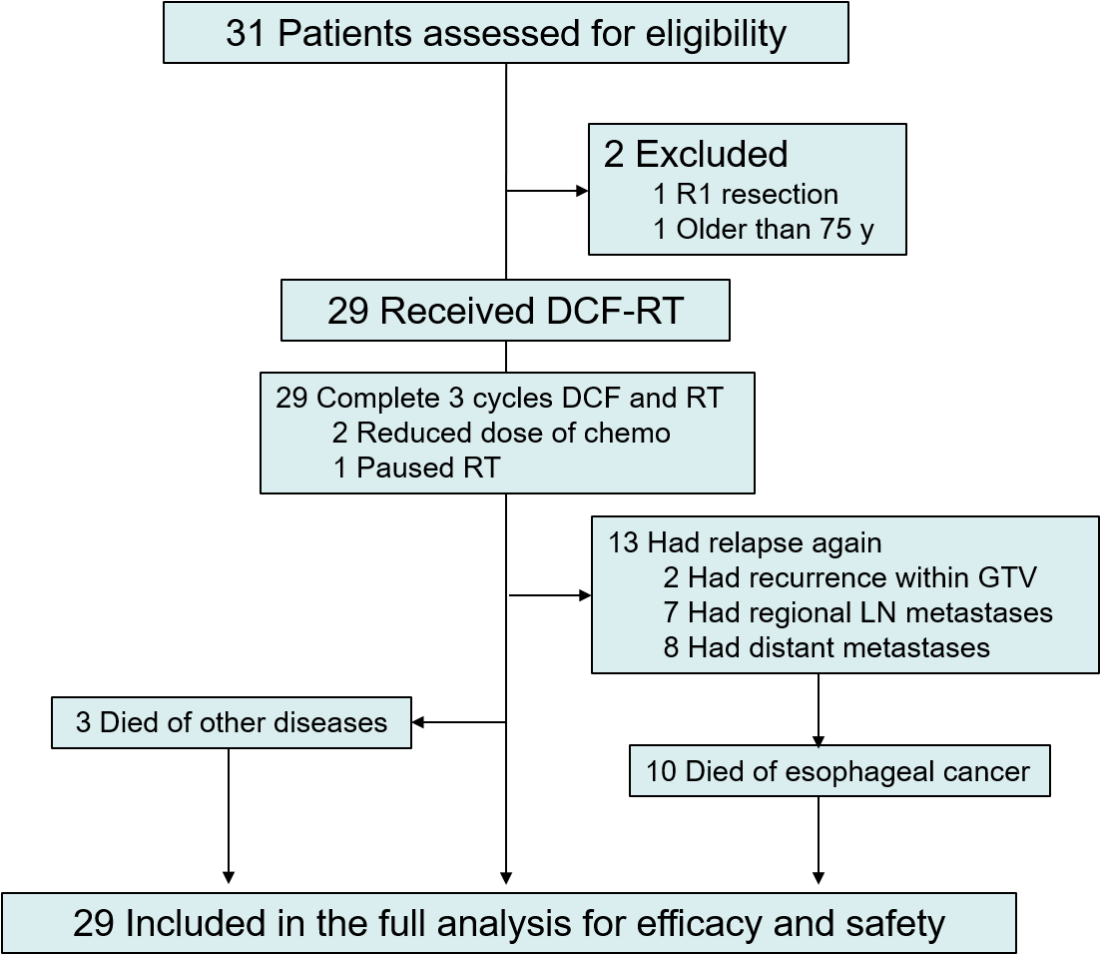
Flowchart of trial participants.

From 2015 to 2023, a total of 29 patients (24 males, 5 females; median age, 64.5 years; age range, 49 to 75 years) were enrolled in this phase II study (**Figure 2**). Patient characteristics are shown in **Table 1**. The median gross tumor volume was 11.4 cm³ (range, 1.4–61.6 cm³). Three patients had grade 4 neutropenia, but radiotherapy was suspended for one day in only one of those patients because the other 2 patients had grade 4 neutropenia after completion of RT. Twenty-eight patients did not pause RT. All patients received 3 cycles of chemotherapy during RT. The rate of completion of this regimen without reduction of the dose of chemotherapy was 89.7%. The median observation period was 77.5 months (IQR, 45.6-89.5 months) for patients who survived. At the last observation date, 13 of the 29 patients had relapse again. Among the 13 patients who experienced progression, only 3 developed recurrence within the original GTV, whereas 7 developed regional lymph node recurrence outside the irradiation field. Distant metastases were observed in 8 patients, including several patients with concurrent locoregional failure. Thirteen of the 29 patients died: deaths were due to progression disease in 10 patients and intercurrent diseases in 3 patients.

**Table 1:** Patients’ characteristics.

Age		years
	median	64.5
	range	49-75
Gender		No.
	male	24
	female	5
Initial pathological stage (UICC* 2009)		No.
	IA-B	7
	IIA-B	7
	IIIA-C	14
	IV	1
Histology		No.
	Squamous cell carcinoma	25
	Basaloid cell carcinoma	1
	Adenocarcinoma	1
	Carcinosarcoma	1
	Adenosquamous carcinoma	1
Disease-free interval between surgery and recurrence		No.
	≤ 12 months	11
	> 12 months	18
Neoadjuvant chemotherapy		No.
	yes	22
	no	7
Recurrent region		No.
	supraclavicular	5
	mediastinal	22
	abdominal	4
	anastomosis	1
Gross Target Volume		cm³
	median	11.4
	range	1.4-61.6
Performance Status (ECOG)		No.
	0	23
	1	6

*UICC: Union for International Cancer Control, †ECOG: Eastern Cooperative Oncology Group.

### 3.2 Efficacy

**Figure 3:**
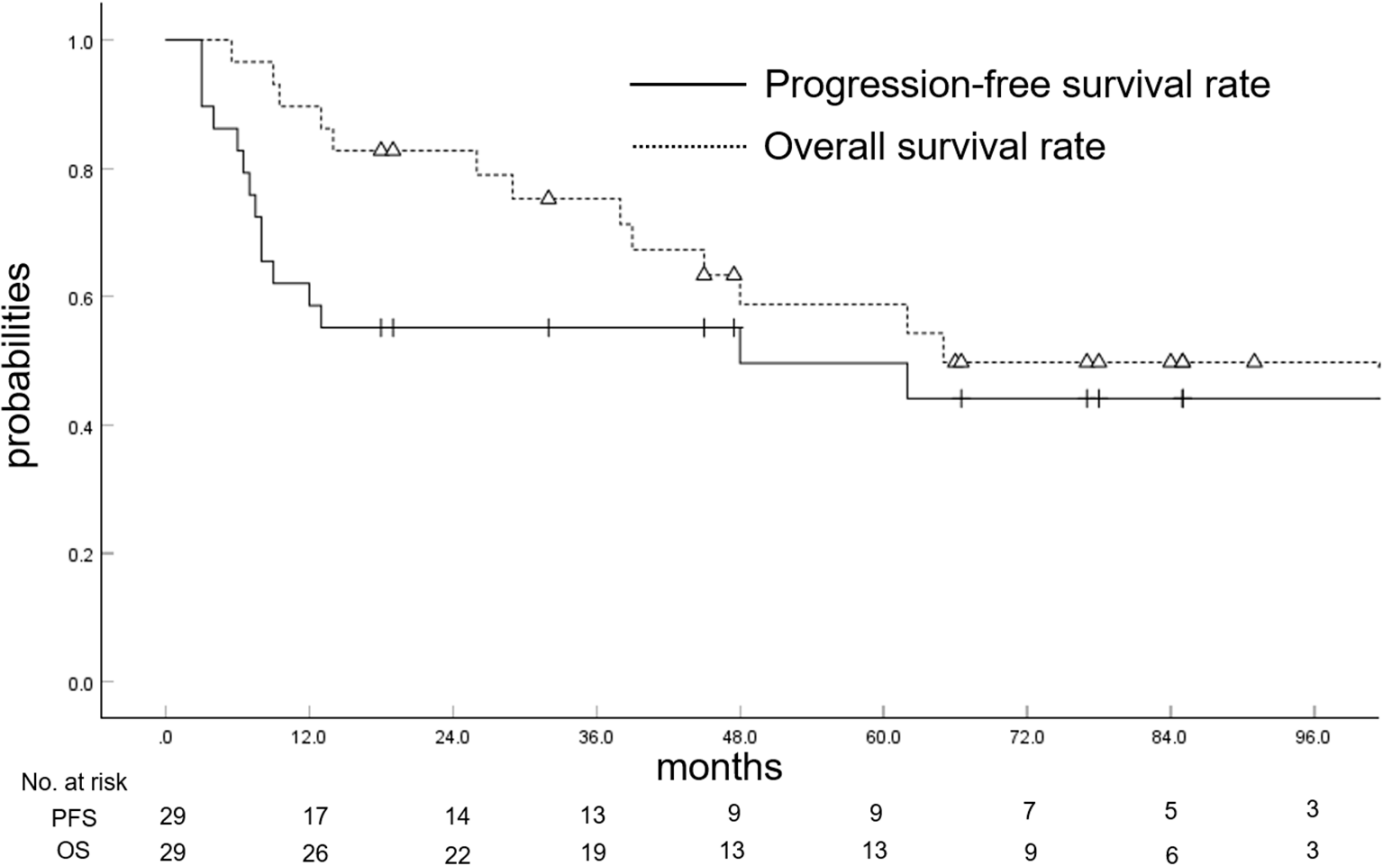
Progression-free survival and overall survival rates in patients with postoperative locoregional recurrent esophageal cancer (Kaplan-Meier method). The 3-year and 5-year progression-free survival rates were 58.8% and 49.7%, respectively, with a median progression-free survival period of 48.0 months. The 3-year and 5-year overall survival rates were 75.2% and 58.6%, respectively.

The 3-year and 5-year progression-free survival rates were 55.2% (95% confidence interval (CI) = 37.1-73.2%) and 49.7% (95% CI = 30.5-68.9%), respectively, with a median progression-free survival period of 48.0 months, substantially exceeding the predefined target value of 24 months (**Figure 3**). The 3-year and 5-year overall survival rates were 75.2% (95% CI = 59.3-91.0%) and 58.8% (95% CI = 39.8-77.8%), respectively (**Figure 3**).

### 3.3 Safety

Adverse events are shown in **Table 2**. A patient had grade 3 heart failure during 3^rd^ cycle of DOC+CDDP+5-FU. The patient discontinued chemotherapy (5-FU only) after the second day of 3^rd^ cycle; however, he completed RT without pause. As adverse events that occurred 3 months or more after completion of chemoradiotherapy, no patient had a grade 2 or higher adverse event, although grade 1 focal pulmonary fibrous changes were often observed.

**Table 2:** Adverse events (n=29).

	Number (%)				
	Grade 1	Grade 2	Grade 3	Grade 4	Grade 5
anemia	6 (20.7)	4 (13.8)	1 (3.4)	0	0
neutropenia	1 (3.4)	7 (24.1)	7 (24.1)	3 (10.3)	0
thrombopenia	7 (24.1)	0	0	0	0
mucositis	13 (44.8)	11 (37.9)	0	0	0
diarrhea	7 (24.1)	1 (3.4)	0	0	0
pneumonitis	5 (17.2)	0	0	0	0
hoarseness	1 (3.4)	0	0	–	–
dermatitis	5 (17.2)	5 (17.2)	0	0	0
fatigue	1 (3.4)	0	0	–	–
nausea	6 (20.7)	3 (10.3)	1 (3.4)	–	–
heart failure	0	0	1 (3.4)	0	0
alopecia	9 (31.0)	5 (17.2)	–	–	–
edema limbs	2 (6.9)	0	0	–	–

### 3.4 Prognostic factor

Of the 29 patients who were enrolled in this study, 21 patients had received neoadjuvant chemotherapy (CDDP + 5-FU) before esophagectomy. There was no significant difference between the progression-free survival rate in patients who received neoadjuvant chemotherapy before esophagectomy and that in patients who did not receive neoadjuvant chemotherapy (p=0.589, HR = 1.419 (95% CI = 0.399-5.038)). Age, performance status, disease-free interval, recurrent site, and GTV were evaluated. According to the log-rank test, there was no other significant prognostic factor for progression-free survival rate.

## 4 Discussion

The aim of this phase II trial was to determine the efficacy and safety of RT combined with DOC, CDDP and 5-FU as a salvage therapy for postoperative locoregional recurrent esophageal cancer. The present study was designed based in part on our previous prospective phase II trial of chemoradiotherapy with nedaplatin and 5-fluorouracil for postoperative locoregional recurrent esophageal cancer (11). In that study, the median progression-free survival was approximately 12 months, which served as one of the historical benchmarks for the current trial. The results indicate that DOC+CDDP+5-FU +RT offers a significant improvement in progression-free survival compared to conventional regimens, with a median progression-free survival period of 48.0 months and a 5-year progression-free survival rate of 49.7% (**Table 3**). These findings suggest that incorporating docetaxel into a standard chemoradiotherapy regimen may provide a more effective treatment strategy for patients with postoperative locoregional recurrent esophageal cancer.

**Table 3:** Literature review of treatment results of chemoradiotherapy for postoperative locoregional recurrent esophageal cancer.

Author	Year	No.	Histology	Methods	Total dose/fraction size	RT method	Median PFS* period	PFS rate
Jingu (11)	2012	30	Squamous cell carcinoma (Sqcc)	CDGP+5-FU+RT	60 Gy/conventional fractions	11 ENRT†, 19 IFRT‡	12 months	29.3% (3-year) 25.1% (5-year)
Zhang (10)	2015	27	Sqcc	CDDP/CDGP+5-FU+RT	46–60 Gy/conventional fractions	ENRT	16.4 months	33.9% (2-year)
Ito (7)	2022	51	Sqcc	CDDP+5-FU+RT	60 Gy/conventional fractions	IFRT	8.2 months	22.9% (3-year)
Chen (6)	2020	48	Sqcc	CDDP+5-FU+RT	50.4–60 Gy/conventional fractions	ENRT	13.9 months	27% (3-year)
The present study	2025	29	25 Sqcc, 1 adenocarcinoma, 3 others	CDDP+5-FU+DOC+RT	60 Gy/30 fractions	IFRT	48 months	55.2% (3-year) 49.7% (5-year)

*PFS, progression-free survival, ^†^ENRT, Elective nodal radiotherapy, ^‡^IFRT, involved-field radiotherapy.

This study included 5 patients with supraclavicular lymph node recurrence. We acknowledge that the classification of supraclavicular lymph node recurrence varies among staging systems. However, supraclavicular lymph node recurrence is often treated with local therapies aimed at curative treatment in esophageal squamous cell carcinoma, and was therefore included in this study, similar to previous studies on salvage chemoradiotherapy.

The results of chemoradiotherapy for postoperative locoregional recurrent esophageal cancer have gradually improved. The reasons given for the improvement are that recurrence has been detected earlier by FDG-PET, that supportive care for treatment has developed and that RT technology has improved. Therefore, the favorable outcomes observed in the present study should be interpreted in the context of temporal advances in staging, treatment delivery, and supportive care. Routine use of FDG-PET may have enabled earlier detection of recurrence, while improvements in radiotherapy planning and supportive care may have contributed to better treatment outcomes. Consequently, the contribution of the DCF regimen itself cannot be completely isolated in this single-arm study. Nevertheless, the magnitude of improvement observed in this study substantially exceeded that generally reported in historical series, suggesting that the DCF regimen may have contributed meaningfully to the observed benefit. Bao et al. reported that DOC + 5-FU +RT was more effective than CDDP + 5-FU +RT for postoperative locoregional recurrent esophageal cancer (5). Taxanes, including DOC, act as not only cytotoxic antitumor drugs by promoting tubulin conjugation and stabilizing microtubule formation, thereby inhibiting mitosis, but also excellent radiosensitizers by arresting the cell cycle in the G2/M phase. Using DOC in concurrent chemoradiotherapy may become the standard in the future. However, toxicities are a problem in concurrent chemoradiotherapy. Especially, there are concerns about severe hematologic adverse events with DOC+CDDP+5-FU +RT; however, it was performed very safely in this phase II study. Although the present regimen incorporated three cytotoxic agents concurrently with RT, the dose intensity of each cycle was lower than that of conventional systemic DOC+CDDP+5-FU regimens used in advanced esophageal cancer. This modified biweekly schedule was designed to balance treatment efficacy and tolerability during concurrent chemoradiotherapy. The reduced incidence of adverse events appears to be due to the use of IFRT. It has been reported that DOC+CDDP+5-FU +RT was performed for newly diagnosed advanced esophageal cancer and many severe hematologic toxicities occurred (13). Elective nodal RT (ENRT) was used in that study. We previously performed a matched-pair analysis for IFRT or ENRT, and the results showed that patients who received IFRT had a significantly better OS (14). Although diagnosis of lymph node metastases is not easy, it is important to use a combination of FDG-PET and the ratio of short axis to long axis. In the present study, LN metastasis recurrences near the irradiation field were observed in several cases. However, even in such cases, salvage RT may be possible again. In this study, chemoradiotherapy could be performed again in 2 patients with marginal recurrence, and one of those patients achieved a durable complete response (CR).

It was shown that even if neoadjuvant chemotherapy had been performed, salvage chemoradiotherapy was effective. CDDP + 5-FU was performed in all the cases. However, in Japan, it was shown DOC+CDDP+5-FU was better than CDDP + 5-FU as neoadjuvant therapy (15), and the number of patients with a history of DOC+CDDP+5-FU treatment should therefore increase in the future (16). However, for patients with recurrence after neoadjuvant DOC+CDDP+5-FU and surgery, salvage chemoradiotherapy showed efficacy (17), and we need to clarify whether DOC+CDDP+5-FU +RT can achieve similar results in such patients in the future.

Since it is difficult to deliver a curative dose to patients who have already undergone neoadjuvant chemoradiotherapy or definitive chemoradiotherapy, other treatments should be considered. Previously, chemotherapy was the only option in such cases, but the long-term prognosis was poor (18). Recently, immunotherapy has shown better outcomes than those of chemotherapy alone when used as an additional treatment after surgery or for treating metastases. To date, no prospective clinical trial has specifically evaluated immune checkpoint inhibitor-based therapy exclusively in patients with postoperative locoregional recurrent esophageal cancer without distant metastasis. Consequently, the optimal role of immunotherapy in this clinical setting remains uncertain, and prospective studies are warranted. Although immune checkpoint inhibitors have become standard in metastatic settings, their role in isolated locoregional recurrence remains unclear. In an exploratory subgroup analysis of patients with locoregional recurrence in CheckMate 648, the median overall survival was approximately 14 months in patients treated with nivolumab plus ipilimumab (19). Because this was a *post hoc* subgroup analysis from a randomized trial with different eligibility criteria and treatment settings, direct comparisons with the present study should be interpreted cautiously. At present, evidence supporting omission of chemoradiotherapy in patients with postoperative locoregional recurrent esophageal cancer who have not received prior radiotherapy remains limited. Liu et al. reported the results of a prospective study in which additional administration of sunitilimab was performed after chemoradiotherapy (20). The role of immunotherapy in postoperative locoregional recurrent esophageal cancer remains to be fully defined. Further studies are warranted to evaluate its efficacy, toxicity profile, and cost-effectiveness in this specific clinical setting. Wu et al. showed that immunotherapy and radiotherapy improved treatment outcomes compared to the outcomes of immunotherapy alone, and concurrent use of immunotherapy with radiotherapy may be recommended for cases in which chemotherapy cannot be used (21). In any case, since the results of the current regimen may be the best achievements for patients with postoperative locoregional recurrent esophageal cancer, DOC+CDDP+5-FU +RT remains a viable and potentially preferable option for postoperative recurrent esophageal cancer. Our results suggested that it is worthwhile to conduct a phase III trial for comparing DOC+CDDP+5-FU +RT and CDDP + 5-FU +RT, which has been the standard strategy.

### 4.1 Limitations

This study has several limitations. First, this study was a single-institution study. Second, the study included a relatively small number of patients. Additionally, the study design did not incorporate a control group receiving conventional chemoradiotherapy, which limits the direct comparison of efficacy between DOC+CDDP+5-FU +RT and other standard treatment regimens.

## 5 Conclusions

The protocol of RT combined with DOC, CDDP and 5-FU is a safe and effective salvage treatment for postoperative locoregional recurrent esophageal cancer. The regimen’s ability to significantly extend PFS, coupled with its manageable toxicity profile, indicates that DOC+CDDP+5-FU +RT may be a superior alternative to conventional treatments.

## Data Availability

The raw data supporting the conclusions of this article will be made available by the authors, without undue reservation.
